# The effects of ultrasonic vibration on riveting quality

**DOI:** 10.1038/s41598-022-17095-1

**Published:** 2022-07-28

**Authors:** Zhendong Xie, Feng Chen, Weikai He

**Affiliations:** School of Aeronautics, ShanDong JiaoTong University, Jinan, 250357 People’s Republic of China

**Keywords:** Mechanical engineering, Aerospace engineering

## Abstract

Ultrasonic vibration can reduce the forming force, decrease the friction in the metal forming process and improve the surface quality of the workpiece effectively. The effects of ultrasonic vibration on riveting quality were systematically studied by numerical simulation and experimental methods. The riveting force, interference, riveting head and microstructure of rivet under different vibration conditions were analyzed, in order to study the influence of the ultrasonic vibration on the riveting process. The study results show that the ultrasonic vibration can reduce the riveting force and decrease the friction. Thus, the flow of rivet material was promoted and the interference and interferometric uniformity were enlarged. Riveting quality was improved, and the improvement effect increased with the increase of amplitude. Compared with the conventional riveting, the relative interference was increased by 27.32% and the shear strength was increased by 17.16%, when the amplitude is 5.77 μm.

## Introduction

Riveted joints are widely used in a variety of industries. In some cases, for instance aircraft assembly, riveting is the single most significant approach to connecting parts. When compared to other, older forms of connection, riveting has a number of advantages: the process is straightforward; riveting equipment is easy to operate; it results in good product quality; and it can be used for complex networks of materials^1^.

At the end of the rivet upsetting process, cold work hardening can significantly increase the yield strength of the rivet material, but reduce its plasticity. This makes it difficult to carry out continuous riveting and the upsetting head can easily produce cracks, leading to inadequate installation quality and poor working conditions^2–4^. Traditional manual hole-making and riveting processes are still widely used in aircraft assembly, limiting production efficiency and quality^5^. There is therefore an urgent need to improve the efficiency and quality of riveting.

Considerable efforts has been devoted to investigating the possible ultrasonic vibration-assisted metal plastic forming in recent decades, and this approach is now widely used in practice, including UV-assisted drawing, stamping, extrusion, etc. Marakawa et al.^6^ applied radial ultrasonic vibration to the die during a wire drawing process. The results showed that this improved the surface quality and it enabled the drawing ratio to be increased. Siegert and Mock^7^ found that the forces involved in wire drawing can be reduced by applying ultrasonically oscillating dies. The decrease in drawing forces serves to extend the potential limits of the forming process. The resonance effects, here, produced a periodic reduction in the drawing force of up to 40%. However, as the drawing velocity increased, the effect upon the drawing force became less pronounced, because the number of oscillations per unit of length decreased. Djavanroodi et al.^8^ investigated the impact of ultrasonic vibrations on equal channel angular pressing (ECAP) and found that, the greater their amplitude, the more the forming load reduced. A 13% reduction in the average force was achieved when ultrasonic vibrations with an amplitude of 2.5 μm at 20 kHz were applied. Faraji et al.^9^ tested the use of ultrasonic vibrations in the ECAP process and found that, by superimposing ultrasonic vibrations, it was possible to increase the uniformity of the strain. Rasoli et al.^10^ studied the influence of longitudinal ultrasonic vibrations on a tube spinning process. Here, experimental results showed that low-power longitudinal ultrasonic vibrations can improve inner surface quality, while high-power ultrasonic vibrations can affect the forming forces and the material escape. They concluded that these changes were a result of contact effects associated with the ultrasonic vibrations. Jimma et al.^11^ improved the limiting drawing ratio in a deep drawing process by applying ultrasonic vibrations. Bunget and Ngaile^12^ investigated the ultrasonic vibration-assisted micro-forming and obtained small parts with high surface quality. The friction between the die and the workpiece was improved by ultrasonic vibration, and the extrusion force was reduced.

As the above sample shows, numerous studies have shown that using ultrasonic vibrations in different plastic metal forming processes can help to reduce the forming force and the friction between the die and the workpiece, and promote the flow of materials. Ultrasonic vibrations render the deformation of material more uniform and improve the formed quality of workpieces. Their use also reduces energy consumption and production costs. In view of these advantages, ultrasonic vibration-assisted riveting has begun to attract interest. To date, this has largely been a feature of patents^13,14^ and there are few experimental studies that explore this potential application of ultrasonic vibration technology. Wang et al.^15^ developed a new and special transverse ultrasonic vibration-assisted riveting (TUVR) system to improve the plasticity and qualification of titanium alloy rivets. Compared with the conventional riveting, the formed driven heads by TUVR are not only suffered from the riveting force and hole boundary constrain, but also the acoustic softening and dragging friction force.

The study reported in this paper therefore set out to systematically study the ultrasonic vibration-assisted riveting process by combining experiments with a finite element analysis. The effects of different vibration conditions on riveting force, interference, the microstructure of the upsetting head, and material flow are examined to derive laws governing the relationship between ultrasonic vibrations and the riveting process. This study is of great potential significance for the development of the process and associated engineering applications.

## Ultrasonic vibration assisted riveting

### Establishing an ultrasonic vibration-assisted upsetting system

**Figure 1:**
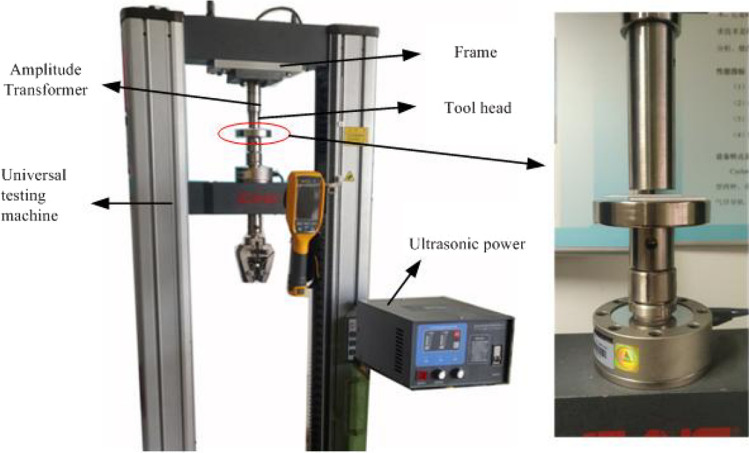
Ultrasonic-assisted riveting test system.

The experimental system used in this study consisted of a universal material testing machine and an ultrasonic vibration unit, as shown in Fig. 1. The maximum testing force of the universal material testing machine was 10 kN. The crosshead speed was 0.01–250 mm/min and its maximum stroke was 750 mm. The ultrasonic vibration unit incorporated an ultrasonic generator, a transducer, an amplitude transformer and a tool head. The transducer in such a unit converts the high frequency electric oscillation generated by the ultrasonic power into mechanical vibration. However, the output amplitude of the mechanical vibration is very small, so it has to be amplified by the amplitude transformer. The ultrasonic vibration then acts on riveting specimens through the tool head. The ultrasonic vibration unit was attached to the experimental machine by means of a frame.

### Test schemes

**Figure 2:**
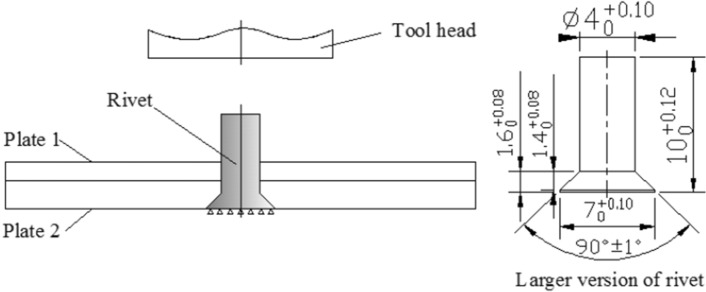
Riveting structure and the size of rivet.

C-riveting and UV-riveting was performed according to specific test schemes, as shown in Table 1. The amplitudes in the table were set value according to the relationship between the amplitude and the output power. Figure 2 shows the riveting structure. The countersunk head rivets (ISO 12281-1999) were made of 6063 aluminium alloy. The plate material was 45 carbon steel, with the size of the upper plate being 50 mm × 50 mm × 2 mm and the size of the lower plate being 50 mm × 50 mm × 3 mm. The core was positioned in the center of the sheet and had a diameter of 4.1 mm. The Ra of the hole's surface was less than 1.6 μm and the Ra of the lower surface was less than 3.2 μm.

**Table 1:** Test schemes.

Number	Power (W)	Frequency (kHz)	Amplitude (μm)	Velocity (mm·min^−1^)	Vibration condition
1	–	–	–	3/30	Without vibration
2	1500	28	3		Applying vibration when the load reaches to 1 kN until the end of the experiment
3			3.6		
4			4.32		
5			4.81		
6			5.78

## The plastic deformation behavior of materials used in an ultrasonic vibration-assisted riveting process

### The effect of ultrasonic vibration on the riveting force

**Figure 3:**
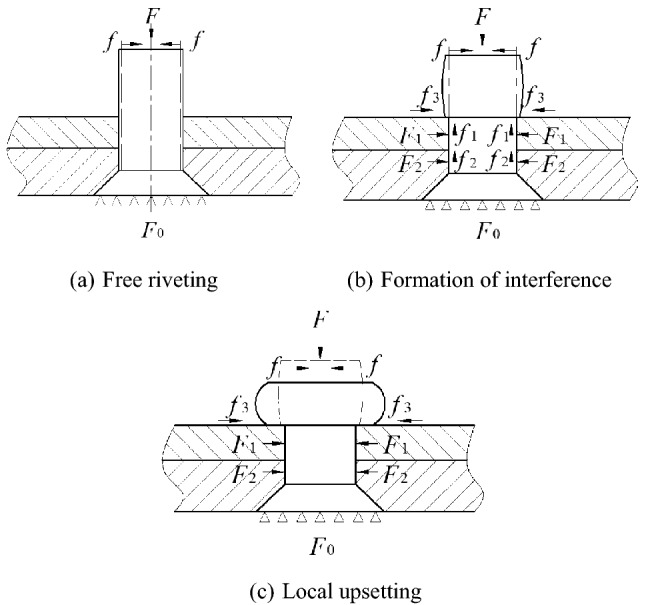
Deformation process of rivet in riveting process.

An ultrasonic vibration-assisted riveting process consists of three stages, as shown in Fig. 3:

#### Free riveting

Elasticity deformation of the rod material can occur at the start of the loading. When the load reaches a certain point, plastic deformation appears.

#### Formation of interference

After the nail rod contacts the hole wall, the nail rod material continues to flow outward as the load increases. The part of the nail rod in the hole is radially constrained and extruded from the hole wall to form an interference connection. In detail, as the load increases, the flow of the nail rod material into the hole gradually reaches a point of saturation and this is when the interference connection is formed. At the same time, the pin material outside the hole produces a radial flow and the upsetting head begins to form. Any deformation after this is mainly concentrated outside of the hole.

#### Local upsetting

The material inside the hole is no longer flowing and the rest is deformed in a radial direction as a result of the axial compression of the tool head and the friction of the riveting plate surface. This continues until the required amount of riveting is reached and a drum upsetting head of a specific size is formed.

**Figure 4:**
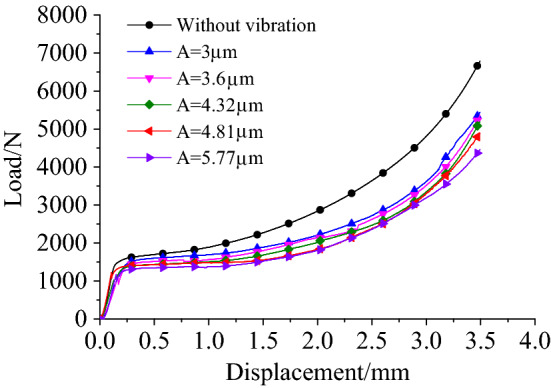
Load–displacement curves under different vibration conditions at 28 kHz frequency.

**Figure 5:**
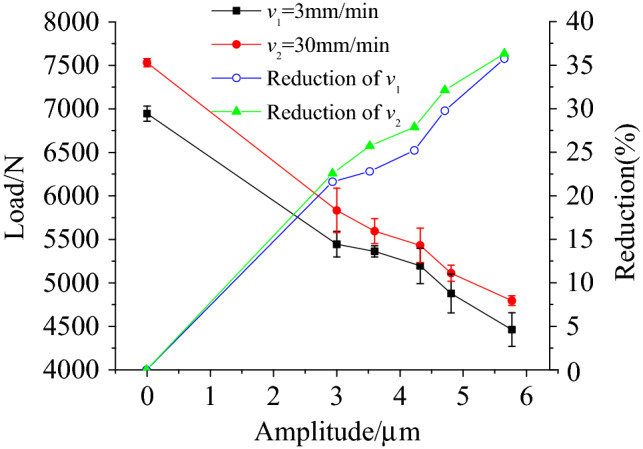
The variation of maximum riveting load and load drop with amplitude under different riveting speed.

Figure 4 shows the load–displacement curves for different amplitudes when the pressure riveting speed was 3 mm/min and the frequency was 28 kHz. The riveting load was reduced under the effect of the ultrasonic vibration. Figure 5 shows the variation of load and how its range decreased as the amplitude increased for 3.5 mm of riveting under two riveting speed conditions. Note that the rate of decrease in the range of the riveting force increased with the increase in amplitude. So, when a specimen was subjected to ultrasonic vibration with an amplitude of 5.77 μm, the load decreased by 2483.01 N (*v* = 3 mm·min^−1^) and 2736.25 N (*v* = 30 mm·min^−1^), respectively, i.e., by 35.75% and 36.33%. Given the same conditions, the specimen with a riveting speed 30 mm·min^−1^ had a larger load. For the two velocity conditions, the load decreased with the increase in amplitude, with the range of decrease being basically the same.

As aluminum alloy is sensitive to strain rates^16^, when the strain rate increased, the plastic deformation could not be fully achieved in the deformation body because of the dislocation caused by the motion, the rotation of the slip surface and the intergranular slip. However, when the strain rate was greater, there was insufficient riveting deformation for the rivet head material to accumulate. Furthermore, the increase in compression speed leads to a decrease in the time of ultrasonic action, so the riveting force decreases less. This caused the overall stress levels to increase.

### The effect of ultrasonic vibration on the shear strength of riveted structure

**Figure 6:**
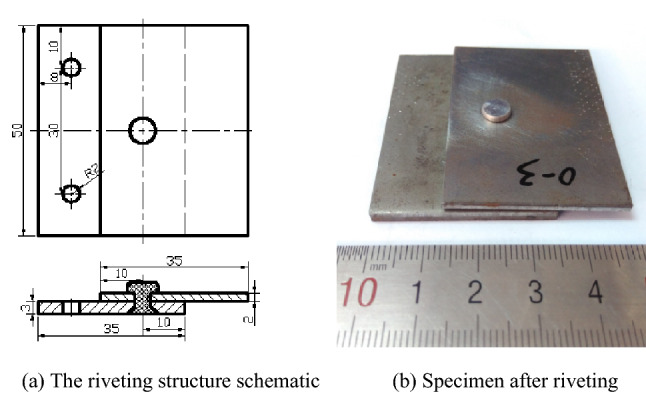
Test structure of shear mechanical properties.

Ultrasonic vibration is effective at reducing the riveting force and has an effect on the riveting quality. Shear strength is one of the key indexes for measuring the mechanical properties of riveted structures. The shear strength of riveted structures under different vibration conditions was therefore also tested. The mechanical shear properties of the riveted structures are shown in Fig. 6. They were tested by the universal testing machine at a shear rate of 2 mm/min.

The shear strength can be obtained using the following formula:

1 $$\tau = \frac{F}{A} = \frac{4F}{{\pi d^{2} }}$$

where, *τ* is the shear strength; *F* is the maximal shear stress; *A* is the cross-sectional area of the rivet; and *d* is the diameter.

**Figure 7:**
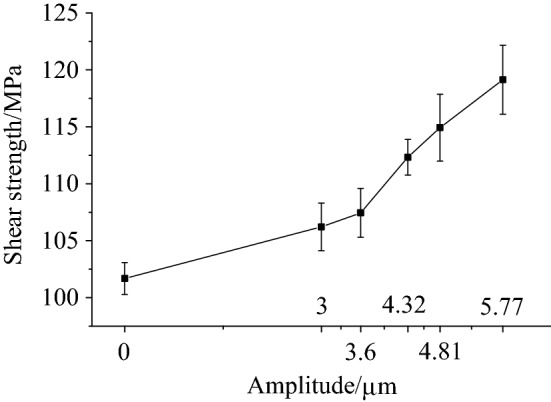
The shear strength under different vibration conditions.

Figure 7 shows the variation in the shear stress according to the amplitude, for a riveting velocity of 3 mm·min^−1^. As the amplitude increased, the shear strength also gradually increased, with the increases in amplitude being 4.45%, 4.67%, 10.47%, 13.04% and 17.16%, respectively. Applying ultrasonic vibrations during the riveting process significantly improves the shear strength of a riveted structure.

After ultrasonic vibration is applied in the riveting process, there is some residual hardening^17,18^, and the hardening of materials contributes to the improvement of shear strength. Ultrasonic vibration promotes the flow of material, increases the diameter of the nail rod, and also improves the shear strength. To properly understand this phenomenon, further studies need to be undertaken regarding the amount of interference and the structure of the riveting material.

### The effect of ultrasonic vibration on the material flow and interference

**Figure 8:**
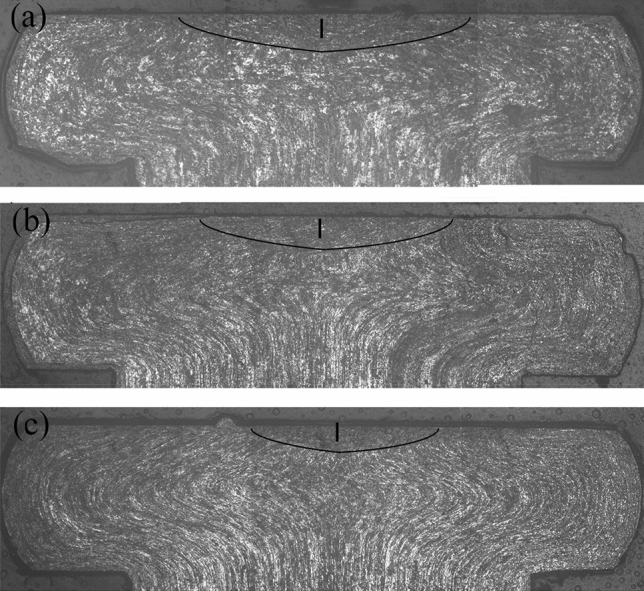
Material flow and stagnant zone of rivet shaft under different vibration conditions: (**a**) without vibration, (**b**) A = 3 μm, (**c**) A = 5.77 μm.

Figure 8 shows the material flow and stagnant zone of the rivet shaft for different vibration conditions. There was little material flow in the stagnant zone (indicated by I in the figure). The area of the stagnant zone was larger when there was no ultrasonic vibration. After the ultrasonic vibration was applied, the friction between the rivet and the tool head decreased due to the surface effect^19^. This reduced the area of the stagnant zone.

**Figure 9:**
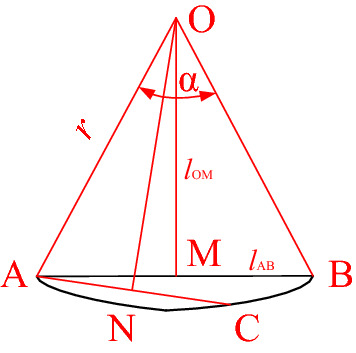
Calculation of the area of stagnant zone.

The stagnant zone is approximately bow-shaped and the angle, $\alpha$ , and radius, r, can be solved by using the method shown in Fig. 9. The formula $S = {{\alpha \pi r^{2} } \mathord{\left/ {\vphantom {{\alpha \pi r^{2} } {360}}} \right. \kern-\nulldelimiterspace} {360}} - {{l_{{{\text{AB}}}} l_{{{\text{OM}}}} } \mathord{\left/ {\vphantom {{l_{{{\text{AB}}}} l_{{{\text{OM}}}} } 2}} \right. \kern-\nulldelimiterspace} 2}$ can then be used to calculate the area. The calculated results for different amplitudes are shown in Table 2, where, *l*_AB_ and *l*_OM_ are the lengths of segments AB and OM, respectively. The area of the stagnant zone decreased when vibration was applied, with the decrease in the range increasing as the amplitude increased. When the amplitude was 5.77um, the area of the stagnant zone had decreased by 51.08%.

**Table 2:** The area of stagnant zone of different conditions.

Amplitude/μm	0	3	4.32	5.77
The area of stagnant zone/mm^2^	0.695	0.517	0.442	0.34

The reduction of the stagnant zone after applying ultrasonic vibrations is due to their surface effect, with more material participating in the deformation. At the same time, the softening effect of the ultrasonic vibrations reduces the material's flow stress and promotes the flow of the nail rod material. This slightly increases the radial size of the nail rod and increases the interference.

Riveting interference is the main technical parameter for measuring riveting quality and has a very important influence on riveting quality evaluations. Interference can be represented in two ways: absolute interference; and relative interference. Absolute interference refers to the difference between the diameter of the nail rod and the size of the initial aperture after deformation. Relative interference is the ratio of the absolute interference to the initial aperture, i.e.:

2 $$I = \frac{{d_{i} - D_{0} }}{{D_{0} }} \times 100\%$$

where, *I* is the relative interference; $D_{0}$ is the initial aperture; and $d_{i}$ is the diameter of the nail rod at different positions after riveting deformation.

**Figure 10:**
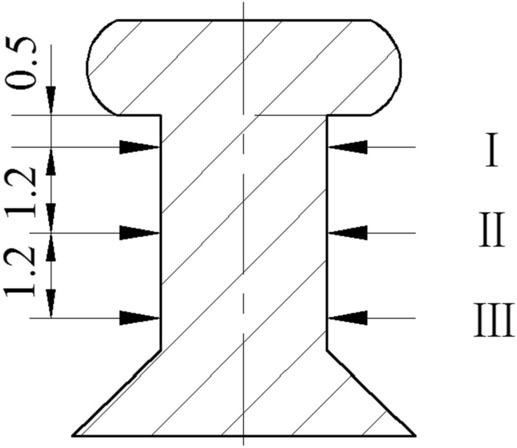
The measuring positions.

In the experiments, the interference was measured by using a longitudinal profile method, with the riveted structure being cut along the axis of the rivet by wire-electrode cutting. The dimensions of three different cross-sections, I, II and III, were measured by an image measuring instrument. The positions of I, II and III are shown in Fig. 10. Table 3 shows the measurement results for different vibration conditions.

**Table 3:** Dimensions of three positions under different amplitude conditions.

Amplitude (μm)	0	3	4.32	5.77
Dimension of position I (mm)	4.195	4.191	4.197	4.196
Dimension of position II (mm)	4.166	4.173	4.183	4.186
Dimension of position III (mm)	4.144	4.156	4.16	4.163

**Figure 11:**
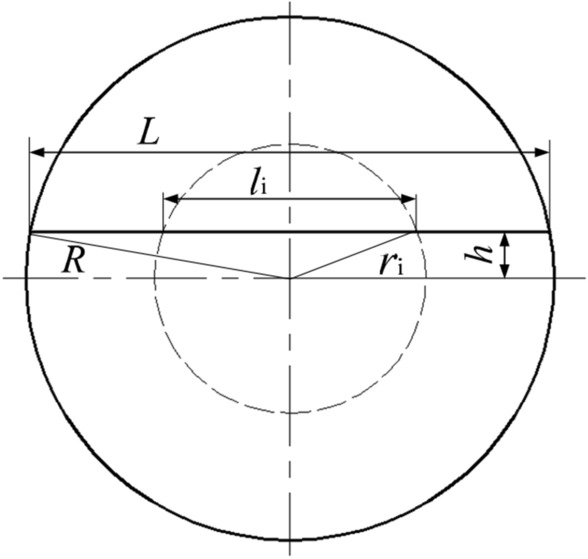
Interferometric compensation.

After the wire-electrode cutting, the cut surface of the rivet shaft deviated from the center. There is therefore a small error between the theoretical measurements and the actual results. This made it necessary to compensate for the deviation in the calculations. The rivets used in the experiment were countersunk rivets, with the diameter of the rivet head being unchanged by the riveting process. In view of this, the diameter of the rivet shaft can be deduced, as shown in Fig. 11, with its actual size being:

3 $$\begin{gathered} d_{i} = 2r_{i} = 2\sqrt {\left( {{{l_{i} } \mathord{\left/ {\vphantom {{l_{i} } 2}} \right. \kern-\nulldelimiterspace} 2}} \right)^{2} + h^{2} } \hfill \\ \;\;\;\;\;\;\;\;\;\;\; = 2\sqrt {\left( {\frac{{l_{i} }}{2}} \right)^{2} + R_{{}}^{2} - \left( \frac{L}{2} \right)^{2} } \hfill \\ \end{gathered}$$

where, $r_{i}$ is the radius of the rivet shaft at different positions; $l_{i}$ is the length of the cross section; *h* is the gap between the cross section and the central surface; $R$ is the diameter of rivet head; and $L$ is the length of the cross section of rivet head, as shown in Fig. 11.

**Figure 12:**
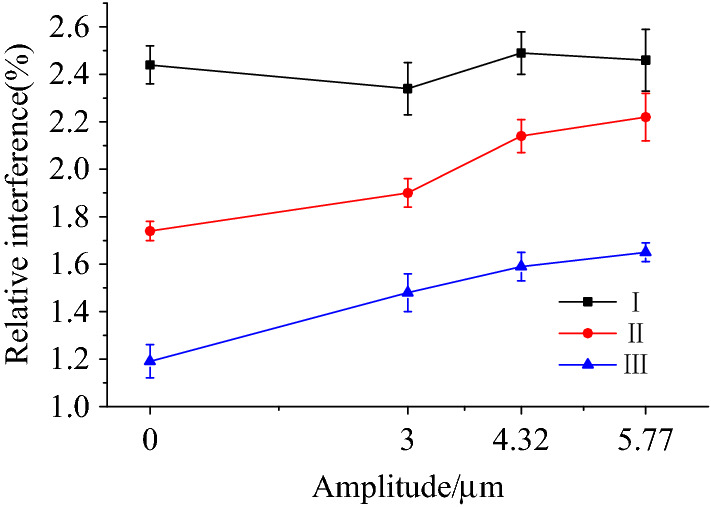
Variation of relative interference with amplitude.

Figure 12 shows the variation in the relative interference in relation to the amplitude at the three cross-section positions (I, II and III). It can be seen that the relative interference for cross-section I was the largest, followed by cross-section II, with the smallest being cross-section III. Due to the large interference at position I, the ultrasonic vibration has little effect on it, the interference value fluctuates due to experimental errors. The effect of ultrasonic vibration on the interference of positions II and III is obvious. When the amplitude was 5.77 μm, the relative interference increased by 27.59% and 38.66%, respectively.

To quantify the uniformity of the interference formed during the riveting process, Eq. (4) was used to calculate the standard deviation coefficient of interference, $\varsigma$:

4 $$\varsigma = \sqrt {\frac{1}{M}\sum\limits_{i = 1}^{M} {\left( {I_{i} - \frac{1}{M}\sum\limits_{i = 1}^{M} {I_{i} } } \right)} } /\left( {\frac{1}{M}\sum\limits_{i = 1}^{M} {I_{i} } } \right)$$

where, *M* is the total number of measured positions; and $I_{i}$ is the relative interference at the ith position. The deformation uniformity of the rivet shaft is denoted by $\varsigma$, and the smaller its value, the less the volatility of the interference. Thus, the interference at different positions is the same when the value of $\varsigma$ is 0. Table 4 shows the standard deviation coefficient of interference uniformity for different amplitudes. The interference uniformity greatly improved after applying ultrasonic vibration. When the amplitude was 5.77 μm, the uniformity of interference increased by 27.32%, when compared to the absence of any vibration. Interference uniformity has an important effect on the fatigue life of a rivet. The greater the interference uniformity, the longer the fatigue life^20^. Thus, applying ultrasonic vibrations during riveting is improves a rivet's fatigue life, and this increases with an increase in amplitude.

**Table 4:** Standard deviation coefficients of interferometry under different amplitude conditions.

Amplitude (μm)	0	3	4.32	5.77
Standard deviation coefficient	0.286	0.184	0.179	0.161

## Finite element analysis of ultrasonic vibration assisted riveting

### Creation of the finite element model

Ultrasonic vibration-assisted riveting can be considered a large dynamic kind of deformation. The explicit dynamic method offered by ABAQUS has strong nonlinear analysis functions. It can automatically select suitable load increments and convergence criteria and continuously adjust these parameters during the analysis process. Many researchers have used finite element software to analyze the riveting deformation process, the results of which serve as a frame of reference^21,22^. ABAQUS was therefore used to further study the deformation behavior of the materials involved in the ultrasonic vibration-assisted riveting process.

**Figure 13:**
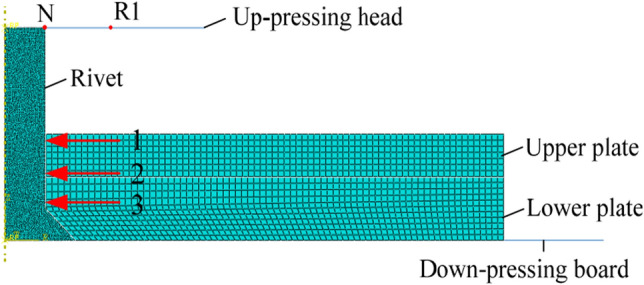
Riveted finite element model.

The experimental conditions described above were used to establish a finite element analysis model. The rivet was treated as an axisymmetric object, so the model could be simplified to a two-dimensional axisymmetric problem, as shown in Fig. 13. The size of the finite element model was the same as the samples used in the experiment. The upper pressure head and the lower roof plate were set as analytically rigid bodies and reference points were created for the upper and lower pressure heads. The parameters of the rivet and connecting plate materials are shown in Table 5.

**Table 5:** Material parameters of 6063 aluminum alloy and 45 steel.

Material	Density (g·cm^−3^)	Elastic modulus (GPa)	Poisson's ratio
6063 Aluminum alloy	2.7	69	0.33
45-Steel	7.85	210	0.269

The analytic process was divided into two steps (Step-1 and Step-2). There was no ultrasonic vibration in Step-1 and the reduction was 1 mm. The variations in the ultrasonic vibrations in Step-2 are shown in Table 6, where the reduction was 2.5 mm. A penalty function contact algorithm was used to define the friction between the different contact surfaces, with the initial friction coefficient being set to 0.15. The boundary conditions of the finite element model were defined according to the experimental conditions, with the lower plate being defined as completely fixed. Similar to the tests, ultrasonic vibration was applied to the upper platen in the numerical model with the same frequency of 28 kHz, by generating a sine wave displacement with the help of periodic type amplitude keyword^21^.

**Table 6:** Finite element analysis scheme.

Number	Frequency (kHz)	Amplitude (μm)	Velocity (mm·min^−1^)	Step-1	Step-2
1	–	–	30	Without vibration	Without vibration
2	28	1		Without vibration	With vibration
3	28	2			
4	28	3			
5	28	4			
6	28	5			
7	28	6			
8	28	7			

### The effect of ultrasonic vibration on the riveting force

**Figure 14:**
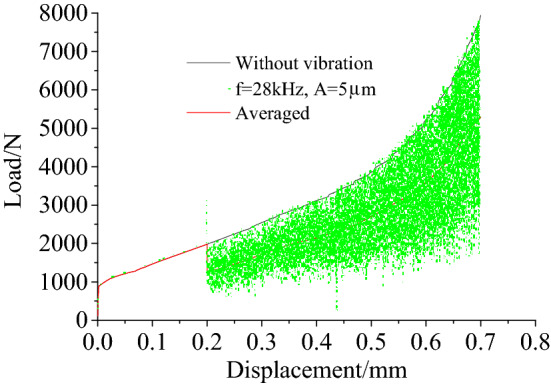
The load–displacement curves.

**Figure 15:**
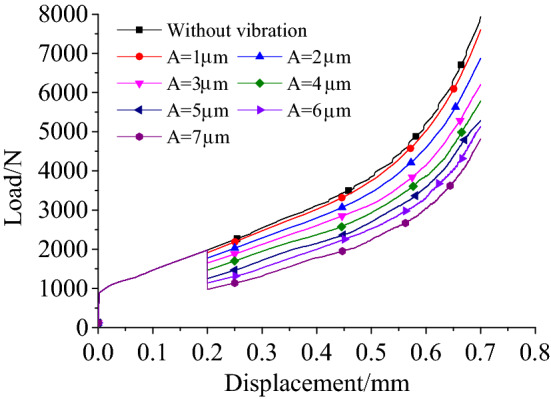
The load–displacement curves under different amplitudes.

Figure 14 shows the load–displacement curves for a riveting speed of 30 mm·min^−1^. The simulation results indicate that the load fluctuates with the displacement when ultrasonic vibration is applied. The fluctuation in the loads is averaged to a single solid red line in the figure. Figure 15 shows the average load-time curves. It can be seen from the figure that the load decreased and that the range of the decrease increased with an increase in amplitude. When compared with the experimental results, there was an immediate decrease in the load and the value of the decrease remained unchanged after the ultrasonic vibration was applied in the simulation. This is because the finite element analysis could only represent the superposition of the ultrasonic vibration, but not the acoustic softening and hardening to be found in actual deformation.

### The effect of ultrasonic vibration on the interference

**Figure 16:**
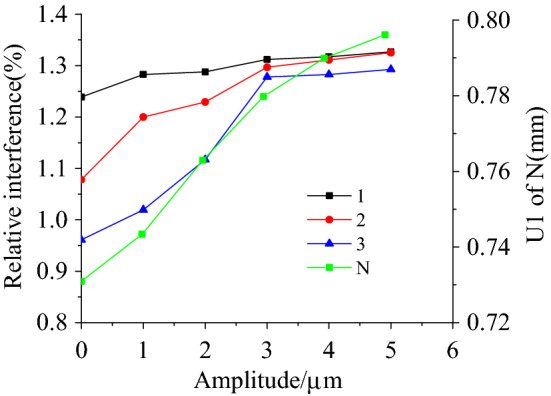
The displacement of node N in x-axis and the relative interference of node 1, 2 and 3 vary with the amplitude.

Figure 16 shows a finite element analysis of the displacement of a node, n, in the x-axis, against variations in the relative interference according to the amplitude at three different positions, 1, 2 and 3. The displacement of node n in the x direction increased after the ultrasonic vibration was applied, indicating that the flow resistance of the material reduced. This implies that the friction between the tool head and the rivet was reduced by the ultrasonic vibration. In addition, the interference at position 1 was largely unchanged by an increase in the amplitude, while the interference at positions 2 and 3 showed an obvious increase. When the amplitude was less than 3 μm, the increase in the interference was especially large.

The material flow at position 1 was adequate and the strength of 6063 aluminum alloy is less than 45 steel. Ultrasonic vibration therefore has little effect on the interference. With regard to the interference between the rivets and connecting plates, the friction between them hinders the downward flow of the rivet shaft materials. If there is no vibration, the high level of friction leads to there being less of a downward flow of material, producing a large difference in the relative interference. After ultrasonic vibrations are applied, the friction is reduced, which is more conducive to the downward flow of the rivet material, making the deformation of the rivet more uniform and elevating the uniformity of the relative interference. As finite element analysis can only express the superposition effect of ultrasonic vibrations, the relative interference value obtained in this way is smaller than the experimental value, but it displays the same basic tendencies.

### The effect of ultrasonic vibration on strain

**Figure 17:**
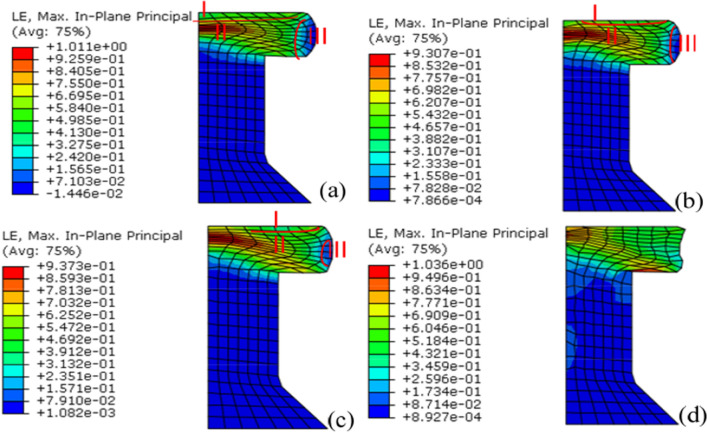
Strain after riveting under different vibration conditions (**a**) no vibration, (**b**) A = 1 μm, (**c**) A = 3 μm, (**d**) A = 5 μm.

Figure 17 shows the finite element analysis results for the effect of ultrasonic vibration on the strain of the rivets. After riveting, the rivet tail has a drum shape, which is similar to free upsetting. In Fig. 17, I, II and III denote a difficult-deformation zone, large-deformation zone and small-deformation zone, respectively. When ultrasonic vibration was not applied, the rivet tail had larger difficult-deformation and small-deformation zones and the rivet tail had an obvious bulge. After the vibration was applied, the difficult-deformation zone and small-deformation zone decreased, while the large-deformation region increased. These results show that the variation in the effective strain tends to become uniform under the effect of ultrasonic vibration. The improvement becomes more evident as the amplitude increases. There was no obvious drum shape when the amplitude was 5 μm and the difference between the deformation zones was no longer obvious, so the deformation was more uniform.

The experimental and finite element analysis results show that the riveting force decreases under the effect of ultrasonic vibration and the scale of this decrease increases with an increase in the amplitude. This is a result of the increase in the amount of interference and evenness of the riveted structure. The strength and fatigue life of the riveted structure are also improved by this.

When applying ultrasonic vibration, its energy is preferentially absorbed by local defects, such as dislocations, voids and grain boundaries. This creates additional stress and can result in dislocations moving more easily, which reduces the activation energy of the material. Thus, the material softens and the flow stress decreases^22,23^. In addition, high frequency ultrasonic vibration increases the activity and temperature of particles in the material, resulting in thermal softening related to crystal dislocation^24^. Under the combined action of superposed static loads, the riveting force decreases^25^.

As a result of applying ultrasonic vibrations, the material begins to soften to a certain extent and its flow stress decreases. The material of the rivet shaft starts to flow relatively easily. This makes the deformation of the rivet shaft more uniform during the riveting process, with the difference in the relative interference at different positions being generally small, improving the shear strength of the riveted structure.

As well as softening the material, ultrasonic vibration also an effect upon the friction between the workpiece and the tool head. There is instantaneous separation between the workpiece and the tool after ultrasonic vibration is applied and the reverse in the friction force vector makes it beneficial to the flow of the nail rod material for a part of the vibration period^26^. The local thermal effect reduces adhesion welding^27^ and improves the machining lubrication^19,28^. As a result of the "surface effect" of the ultrasonic vibrations, the friction between the rivet head and the tool head, rivet shaft and plate are reduced. The decrease in the friction force can also promote the flow of the nail rod material.

## Conclusions

This study examined the influence of ultrasonic vibration on the riveting process of 6063 aluminum alloy by analyzing the pressure riveting force, relative interference, shear strength of the riveted structure and the material flow. Both physical experiments and a finite element analysis were undertaken. The principal conclusions are as follows:

1. Ultrasonic vibration causes a softening effect, which reduces the riveting force at different riveting speeds. The scale of the decrease in the riveting force increases with an increase in the amplitude of the vibrations.
2. The friction between contact surfaces decreases when ultrasonic vibration is applied. This promotes the material flow of the rivet shaft. The area of zones where deformation is difficult decreases, while the area of large deformation zones increases. The larger the amplitude, the more obvious the reduction of friction between the contact surfaces and the smaller the area of the difficult deformation zone.
3. Ultrasonic vibration has both a softening effect and a surface effect. It makes the deformation of the rivet shaft more uniform, increases the quantity of interference and improves the evenness of the riveted structure. Together, these attributes help to improve the riveted structure's shear strength and fatigue life.

### Consent for publication

The author agrees to publication in the Scientific Reports and confirms that the work described has not been published before (except in the form of an abstract or as part of a published lecture, review, or thesis), and its publication has been approved by all co-authors.

## Data Availability

The data sets supporting the results of this article are included within the article.

## References

[1] Yoon TH, Kim SJ (2011). Refined numerical simulation of three-dimensional riveting in laminated composites. J. Aircr..

[2] Cheraghi SH (2008). Effect of variations in the riveting process on the quality of riveted joints. Int. J. Adv. Manuf. Technol..

[3] Szolwinski MP, Farris TN (2000). Linking riveting process parameters to the fatigue performance of riveted aircraft structures. J. Aircr..

[4] Choo VKS, Reinhall PG, Ghassaei S (1989). Effect of high rate deformation induced precipitation hardening on the failure of aluminium rivets. J. Mater. Sci..

[5] Ni J, Tang WC, Xing Y (2018). Assembly process optimization for reducing the dimensional error of antenna assembly with abundant rivets. J. Intell. Manuf..

[6] Murakawa M, Jin M (2001). The utility of radially and ultrasonically vibrated dies in the wire drawing process. J. Mater. Process. Technol..

[7] Siegert K, Möck A (1996). Wire drawing with ultrasonically oscillating dies. J. Mater. Process. Technol..

[8] Djavanroodi F, Ahmadian H, Koohkan K (2013). Ultrasonic assisted-ECAP. Ultrasonics.

[9] Faraji G, Ebrahimi M, Bushroa AR (2014). Ultrasonic assisted tubular channel angular pressing process. Mater. Sci. Eng. A.

[10] Rasoli MA, Abdullah A, Farzin M (2012). Influence of ultrasonic vibrations on tube spinning process. J. Mater. Process. Technol..

[11] Jimma T, Kasuga Y, Iwaki N (1998). An application of ultrasonic vibration to the deep drawing process. J. Mater. Process. Technol..

[12] Bunget C, Ngaile G (2011). Influence of ultrasonic vibration on micro-extrusion. Ultrasonics.

[13] Toh, C.H. *Ultrasonic Riveting Tool and Method: U.S. Patent 9321099*. (2016).

[14] Götzelmann, J., & Salzmann, H. *Method for Connecting Thermoplastic**, **Coated Components and Plastic Component: U.S. Patent 9821539*. (2017).

[15] Wang X, Qi Z, Chen W (2021). Study on the effects of transverse ultrasonic vibration on deformation mechanism and mechanical properties of riveted lap joints. Ultrasonics.

[16] Wei Q, Cheng S, Ramesh KT, Ma E (2004). Effect of nanocrystalline and ultrafine grain sizes on the strain rate sensitivity and activation volume: fcc versus bcc metals. Mater. Sci. Eng. A.

[17] Xie Z, Guan Y, Yu X, Zhu L, Lin J (2018). Effects of ultrasonic vibration on performance and microstructure of AZ31 magnesium alloy under tensile deformation. J. Central South Univ..

[18] Yao Z, Kim G, Wang Z (2012). Acoustic softening and residual hardening in aluminum: Modeling and experiments. Int. J. Plast..

[19] Xie Z, Guan Y, Zhu L, Zhai J (2018). Investigations on the surface effect of ultrasonic vibration-assisted 6063 aluminum alloy ring upsetting. Int. J. Adv. Manuf. Technol..

[20] Croccolo D, De Agostinis M, Ceschini L, Morri A, Marconi A (2013). Interference fit effect on improving fatigue life of a holed single plat. Fatigue Fract. Eng. Mater. Struct..

[21] Zhuang X, Wang J, Zheng H, Zhen Z (2015). Forming mechanism of ultrasonic vibration assisted compression. Trans. Nonferrous Met. Soc. China..

[22] Siddiq A, Sayed TE (2012). Ultrasonic-assisted manufacturing processes: Variational model and numerical simulation. Ultrasonic..

[23] Siddiq A, Sayed TE (2011). Acoustic softening in metals during ultrasonic assisted deformation via CP-FEM. Mater. Lett..

[24] Langenecker B (1963). Effect of sonic and ultrasonic radiation on safety factors of rockets and missiles. AIAA J..

[25] Wang C, Liu Y, Guo B, Shan D, Zhang B (2016). Acoustic softening and stress superposition in ultrasonic vibration assisted uniaxial tension of copper foil: Experiments and modeling. Mater. Des..

[26] Hung J, Tsai Y, Hung C (2007). Frictional effect of ultrasonic-vibration on upsetting. Ultrasonics.

[27] Daud Y, Lucas M, Huang Z (2006). Superimposed ultrasonic oscillations in compression tests of aluminium. Ultrasonics.

[28] Kumar VC, Hutchings IM (2004). Reduction of the sliding friction of metals by the application of longitudinal or transverse ultrasonic vibration. Tribol. Int..

